# Depressive self-focus bias following failure: an eye-tracking study among individuals with clinical depression

**DOI:** 10.3389/fpsyt.2024.1459831

**Published:** 2024-10-01

**Authors:** Jean Monéger, Ghina Harika-Germaneau, Nematollah Jaafari, Damien Doolub, Laura Warck, Leila Selimbegović, Armand Chatard

**Affiliations:** ^1^ Department of Psychology, University of Poitiers, Poitiers, France; ^2^ Research Center on Cognition and Learning, National Centre for Scientific Research (CNRS) 7295, Poitiers, France; ^3^ Clinical Research Unit, Centre Hospitalier Laborit, Poitiers, France

**Keywords:** depression, eye-tracking, self-focus, failure, attentional bias

## Abstract

**Objective:**

Depression is often characterized by a persistent sense of failure. Cognitive theories of depression suggest that depressed individuals may exhibit a maladaptive cognitive style, characterized by increased self-focus following personal failure. The validity of this proposition, however, is yet to be fully examined. This study aimed to identify the relation between symptoms in major depressive disorder and increased self-focus in failure situations.

**Methods:**

This clinical study involved a cohort of 30 patients diagnosed with and treated for depression. We used an eye-tracking paradigm to observe and analyze gaze direction – indicative of either self-focus or self-avoidance – after remembering a significant failure event.

**Results:**

Contrary to the maladaptive cognitive style hypothesis, a majority of the depressed participants demonstrated an inclination towards self-avoidance following failure. Nevertheless, approximately 30% of the patient group – those with the highest scores of guilt, punishment, and self-blame – displayed a self-focused attentional bias post-failure.

**Conclusions:**

The presence of a maladaptive self-focusing style may be confined to severely depressed patients with high levels of guilt, punishment, and self-blame. These findings could have substantial clinical implications, as attention bias modification interventions could be particularly beneficial for this subgroup of patients.

## 1 Introduction

Cognitive theories suggest that a variety of systematic cognitive biases are integral to the onset and the perpetuation of depressive symptoms (1–7). These cognitive biases in depression, such as the bias towards negative self-referential information, are well documented mechanisms reinforcing maladaptive negative self-schemas (8–11). This paper focuses on one specific bias: the depressive self-focus bias – a tendency to engage in prolonged self-focused attention after experiencing personal failure (12).

### 1.1 Failure-related cognitive bias in depression

Beck’s (1) cognitive theory of depression suggests that, unlike non-clinical populations, individuals with depression harbor negative self-views (self-schemas) and might be especially inclined to process information that sustains these negative self-views. A considerable body of literature has demonstrated that individuals with depression tend to exhibit an attentional bias toward negative information, thus reinforcing the negative schemas outlined in Beck’s theory of depression (see 13 for a review; although see 14). As an example, Hindash and Amir (15) asked participants to indicate whether sentences (e.g., “You get a new job”) were related or not to a word presented afterward. Some words were negative (“Unqualified”) while other where not (“Qualified”). They observed that dysphoric participants were faster to identify negative words as being related to the situations than non-dysphoric participants. This finding underlines a bias toward negative interpretations of situations, emphasizing that depressed individuals tend to favor evaluations consistent with negative self-views.

In this vein, several studies reported that when confronted with failure, depressed individuals produce dysfunctional attributions likely to foster self-blame, such as characterological (e.g., blaming one’s self) rather than behavioral (blaming one’s specific behavior) or circumstantial attributions (e.g., blaming specific circumstances; 16–18). Depression is also characterized by an attentional bias towards negative information, a pattern corroborated by eye-tracking studies (13, 19–26).

Importantly, depression is often associated with aversive self-awareness, which might exacerbate a sense of failure and cause biased self-perception (27–31). Accordingly, it was suggested that depressed individuals may display a maladaptive pattern of focusing their attention inwards in negative situations (12, 32). Specifically, Pyszczynski and Greenberg (12) showed that after false negative feedback on a task allegedly measuring intelligence, subclinically depressed participants, but not non-depressed individuals, preferred to be exposed to a mirror rather than avoid it, indicating a preference for self-focusing stimuli upon failure. This finding provides support for a depressive self-focus bias in individuals suffering from mild (subclinical) depressive symptoms. Understanding self-focus biases in failure context among depressed patient is critical for developing targeted interventions. Identifying such bias could inform more effective therapeutic approaches, such as cognitive-behavioral therapies specifically tailored to address these biases. Yet, there is a lack of empirical findings indicating how such self-focus bias in failure situations might characterize clinical samples, and to what extent depression severity might be associated to this bias. As such, it is crucial to extend previous findings from subclinical to clinical populations (see 33).

### 1.2 The present study

To further address the question of how depressive individuals deploy their attention upon failure, we relied on an eye-tracking paradigm. Eye-trackers are commonly employed to study how individuals direct their visual attention toward prominent elements in their surroundings (e.g., 34, 35). Consequently, they are useful tools for evaluating visual attention, particularly in relation to self-focused attention (36–39). In a recent study, Monéger et al. (40) have shown that participants focused less on their screen-reflected faces after a failure manipulation (indicating self-focus avoidance) than in a control condition. A similar paradigm was used here as it provides a precise, yet subtle and unobtrusive measure of whether individuals deploy their attention toward or away from the self upon failure. In the present study, this paradigm was applied for the first time in a sample of clinically depressed individuals. We would consider the maladaptive bias hypothesis to be supported if individuals with depression exhibit heightened self-focus following failure in comparison to their baseline (prior to the induction of failure).

## 2 Method

The study was conducted between December 2021 and June 2023. Ethical clearance was obtained from the Institutional Review Board of CPP Ouest I (number: 2021-A01098-33), and trial registration was completed at the Clinical Trial Registry before the study began (ClinicalTrials.gov Identifier: NCT0546550). All patients provided written informed consent after a full description of the study. Thirty participants (24 women and 6 men, *M_Age_* = 38.72, *SD_Age_* = 13.33) were recruited from the local University Hospital. All participants were diagnosed with severe major depression (based on an expert diagnosis and confirmed using the MINI-IV). With this sample size, an effect size of *d_z_* = .53 can be detected with a statistical power of 80% in a paired sample t-test.

### 2.1 Patient selection

To participate to this study, participants’ depression diagnosis had to be confirmed 24 hours before the experimental manipulation. The diagnosis was made by a professional clinician using the MINI-IV (see 41). Ineligible participants for the study included individuals with mental deficiencies (IQ < 70), neurological impairments (epilepsy, encephalopathy, head trauma), those forced to stay in the hospital and/or not having healthcare. Eligible participants were required to be aged between 19 and 60 years old, be native French speakers, and have a normal or corrected-to-normal eyesight. Participants wearing rigid lenses were not included in the study to avoid difficulties with the eye-tracker task. All participants were diagnosed with major depression using the MINI-IV (see 41). Patients were diagnosed with unipolar depression (72.41%), bipolar depression (13.79%), or isolated depression (13.79%). As expected, both MADRS (*M* = 27.34, *SD* = 8.23) and BDI scores (*M* = 29, *SD* = 12.42) were high, indicating that participants were characterized with moderate (cut-of at 20 for the MADRS and 21 for the BDI) to severe depression (cut-off at 35 for the MADRS, and 31 for the BD1).

### 2.2 Material availability

Anonymized data, analysis codes, materials, and supplementary analyses are available on the OSF webpage of this study (https://osf.io/94y7v/).

### 2.3 Materials

#### 2.3.1 Failure manipulation

In order to manipulate feelings of failure, we used an autobiographical task. Participants were asked to recall a significant personal failure. They were then instructed to describe the memory details in written, in a similar approach to an autobiographical Memory Tasks (42). To do that, they were guided with specific instructions designed to elicit a vivid memory (e.g., “*Describe in the most detailed manner how you felt and what you thought of during this episode*”, “*How did you feel from a physiological standpoint during this episode*”, etc., for similar tasks see, 43, 44, study 2; 45). After answering these questions, participants completed 4 Likert scale items used as controls (e.g., “The memory I recalled was clear in my mind”, “The memory I described relates to a painful event in my life”, “During this task, I felt in a failure situation”, and “During this task, I was able to relive the emotions I felt during the episode I recalled”), using a 10 point scale ranging from 1 (Strongly Disagree) to 10 (Strongly Agree; the full material is available on the OSF webpage of the project, see anonymized OSF link).

#### 2.3.2 Attentional bias measure

We measured attentional bias in self-focus by using an eye-tracker combined with a reflexive screen (iMac, 27”, 44.5 cm × 65 cm or 17.5” × 25.6” or 1440 × 2560 pixels). The eye-tracker used was an Eye-Link Portable Duo with a sample rate of 500Hz. The experimenters were concealed from the participant (see **Supplementary Online Material** for a photograph of the experimental set-up). An Area Of Interest (AOI) was defined in the center of the screen as a large oval area covering 875,824.98 pixels (i.e., 23.76% of the total screen area). The size of the AOI was similar to the one used in Monéger et al. (40). The experimenter asked patients at the end of each session to gaze at the contour of their screen-reflected faces, thus ascertaining that their reflections were indeed captured in the defined AOI (see “AOI_def.docx” on the OSF webpage). Participants were instructed to complete a lexical decision task: strings of letters were displayed randomly in one of the four corners of the screen, and participants had to indicate as fast and as correctly as possible whether these targets were words (e.g., TABLE) or non-words (e.g., TEBLA). Target words were displayed until the participant provided a response using a button box. To indicate that the target was a word, they had to press a green button on the far right of the button box, and to indicate that the target was a non-word, they had to press a red button on the far left of the button box. During this task, we recorded gaze behavior occurring between the participant’s response and the onset of the next target (i.e., during the inter-trial intervals). Inter-trial intervals durations were randomly selected in a sample of possible duration ranging from short (325 ms) to long (8485 ms, see Footnote 1). Each block of the study used the same inter-trial times so that they were balanced. This range of inter-trial intervals was the same as the one used by Monéger et al. (40). The rationale behind this range is that it promotes a sense of unpredictability that should foster participants’ engagement in the task.

As in previous research, we assessed the total sum of the number of fixations in the AOI that were preceded by a fixation outside the AOI in all the inter-trial intervals for each block (hereafter, saccades in the AOI). Average time spent in the AOI during the inter-trial intervals (hereafter, dwell time) was also assessed, but previous studies using this protocol failed to detect an effect of failure on this measure (see 40). Dwell time and number of saccades in the AOI are negatively correlated: the more saccades toward the self, the less time we spent on average in the AOI (and vice-versa). Indeed, if an individual spent the whole task looking at oneself, this would result in a maximal dwell time, but a minimal number of saccades toward the self. Conversely, a large number of saccades toward the self implies that the participant was not consistently fixated on their reflection. Whereas dwell time reflects ‘time spent looking at the self-reflected face’, the number of saccades toward the self would reflect the ‘number of time someone glanced toward the self after a period of non-focused state’. Given that previous research using the same paradigm identified the number of saccades in the AOI, but not the dwell time spent on the AOI, as a relevant indicator of self-focus bias (40), we report this variable as our criterion for identifying self-focus avoidance. Results regarding average dwell time are reported in the **Supplementary Material**, but should be interpreted cautiously given the negative relation that this indicator maintains with number of saccades into the AOI.

#### 2.3.3 Montgomery-Asberg depression rating scale

The MADRS is a 10 items semi-structured interview scale administered by a trained professional. It assesses changes in core depression symptoms severity such as sleep disturbances, sadness, or suicidal thoughts. In our sample, the internal consistency of the MADRS was acceptable (Cronbach alpha = .76).

#### 2.3.4 Beck depression inventory

The BDI is a self-administered scale including 21-items measuring a broad range of depression symptoms. Each item measure a specific symptom and consists in a set of 4 propositions from which the participant must choose (e.g., Self-Hatred item: “I don’t feel disappointed in myself”, “I am disappointed in myself”, “I am disgusted with myself”, “I hate myself”). In our sample, the scale was associated to a very satisfying internal consistency (Cronbach alpha = .91).

### 2.4 Procedure

During the inclusion session, a professional clinician ensured that the inclusion criteria were met at least 24 hours before the experimental session. In addition to the MINI-IV that provided a diagnosis of depression (among other clinical diagnoses), depressive symptomatology was assessed using the MADRS (a clinician-rated 10 items scale with scores ranging from 0 to 60; 46) and the Beck Depression Inventory (A self-rated 21 items scale with scores ranging from 0 to 61; 47).

During the experimental session, participants were briefed about the eye-tracker and the procedure. Before the completion of the self-focus avoidance measure, participants completed measures of shame- and guilt-proneness. Because these measures are outside the scope of the current article, results relating to these measures in the **Supplementary Material**. They were then asked to stay steady on a chinrest while performing the cognitive task. After a training block of 12 trials, the participants performed a first block of 36 trials. Then, they were asked to complete the failure manipulation. After completing the failure manipulation, they performed a second block of 36 trials of the self-focus avoidance measure.

We conducted a paired sample t-test to investigate the self-focused attentional bias (i.e., more saccades toward the self after vs. before the manipulation), Pearson correlations to assess how depressive symptoms correlate with the self-focused attention after vs. before the manipulation, and subsample analyses comparing patients displaying a self-focused attentional bias (i.e., more saccades toward the self after vs. before the manipulation), and patients who did not. Analyses were performed using R (see OSF webpage for codes).

## 3 Results

### 3.1 Description of the current sample

Because the eye-tracker abruptly stopped functioning during one of the experimental sessions, data from one participant was lost, leaving a sample of 29 depressed patients (23 women and 6 men, *M_age_* = 38.72, *SD_age_* = 13.33).

In addition to a diagnosis of depression, the sample was additionally characterized by comorbid disorders, with some participants diagnosed with generalized anxiety (51.7%), bulimic disorders (13.8%), psychosis (6.9%), obsessive compulsive disorders (17.2%), social phobia (58.6%), panic disorders (during lifetime, 13.8%, and current 24.1%), alcohol (17.2%) and other substance addiction (6.9%), PTSD (20.7%), agoraphobia (58.62%), and mood disorder with psychotic characteristics (10.34%).

Importantly, most of the participants had pharmacological treatment. Thus, 86.21% of the total sample used antidepressants. Other pharmacological treatments included antipsychotics (37.93%) and thymoregulators (17.24%).

Most patients attempted to commit suicide in the past (69%) with a total average number of attempts of 1.76 (*Minimum* = 0, *Maximum* = 5, *SD* = 1.68).

### 3.2 Main results

Regarding the effectiveness of the failure manipulation, using 10-points scales, participants evaluated that their failure recall was clear and precise (*M* = 8.14, *SD* = 2.23), related to a painful event (*M* = 8.41, *SD* = 2.15), produced a sense of failure (*M* = 8.28, *SD* = 2.71) and that they felt they were able to relive the emotions of the situation (*M* = 7.07, *SD* = 2.90).

**Figure 1:**
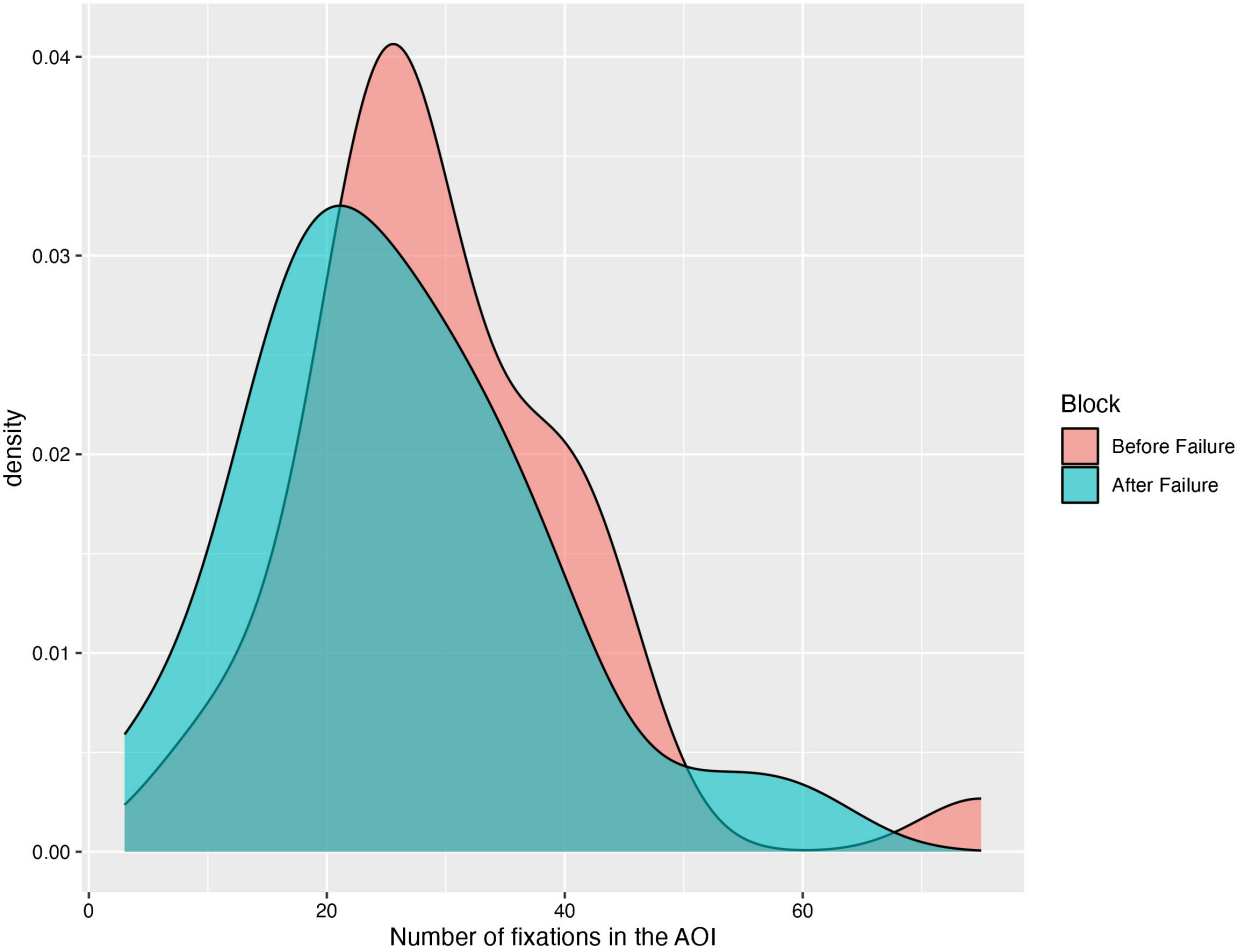
Density plot of saccades in the AOI before and after the failure manipulation.

**Figure 1** illustrates the density of saccades within the AOI (containing the participant’s reflected self-image on the screen) before and after the failure manipulation. Overall, participants displayed fewer saccades within the AOI after recalling a failure memory, compared to before (*M* = 26.00, *SD* = 12.67 and *M* = 29.86, *SD* = 12.52, respectively). A paired-sample t-test has shown this difference to be significant, *t*(28) = 2.42, *p* = .023, Cohen’s *d* = 0.45, 95% CI[0.06, 0.84]. This result contradicts the depressive self-focus attention bias hypothesis. However, it aligns with the pattern of self-focus avoidance observed in previous research involving non-depressed individuals (40).

To further understand the effects observed in this study, we conducted additional analyses. Specifically, we examined whether the increase in self-focus after the failure manipulation correlated with the severity of self-reported depressive symptoms (as indicated by BDI scores). The overall BDI score had a positive but non-significant correlation with increased self-focus after failure, as indicated by a Pearson’s correlation between BDI scores and the difference in saccades toward the self after versus before failure recall, *r*(27)= .31, *p* = .11, 95%CI [-.068,.60] (but see footnote 2). Because the lack of significance may be due to the fact that some depressive symptoms assessed in the BDI are not directly relevant to the experience of failure (i.e., irritability, indecisiveness, loss of appetite, etc.), we conducted further correlation analyses on each BDI depressive symptoms to see whether specific symptoms predicted self-focus after failure (see 48). Moderate to strong correlations with self-focus after the failure manipulation and specific items of the BDI were observed. Specifically, self-focus bias was predicted by Feeling sad (Item 1), Hopelessness about the future (Item 2), Feeling guilty (Item 5), Feeling punished (Item 6), Self-hatred (Item 7), Self-blame (Item 8), Efforts (Item 15), Fatigue (Item 17, see **Table 1**). Importantly, self-focus bias after failure appeared to correlate with a pool of items related to failure and self-blame (i.e., Feeling guilty, Feeling punished, Self-hatred, and Self-blame).

**Table 1:** Correlations between self-focus attentional bias and specific depressive symptoms.

Self-focus attentional bias			
*Depressive symptoms*	*r*	*p*	*95% CI*
1. Feeling sad (*I am so sad and unhappy that I can’t stand it*)	**.42**	.023	[0.062, 0.68]
2. Hopelessness about the future (*I feel the future is hopeless and that things cannot improve*)	*.36*	.051	[-0.0017, 0.65]
3. Feeling like a failure (*I feel I am a complete failure as a person*)	.15	.45	[-0.23, 0.49]
4. Dissatisfaction and Boredom (*I am dissatisfied or bored with everything*)	.31	.10	[-0.065, 0.61]
5. Feeling guilty (*I feel guilty all of the time*)	**.46**	.013	[0.11, 0.71]
6. Feeling punished (*I feel I am being punished*)	**.42**	.024	[0.060, 0.68]
7. Self-hatred (*I hate myself*)	**.46**	.012	[0.11, 0.71]
8. Self-blame (*I blame myself for everything bad that happens*)	*.34*	.068	[-0.026, 0.63]
9. Suicidal desires (*I would kill myself if I had the chance*)	.30	.11	[-0.075, 0.60]
10. Wanting to cry (*I used to be able to cry, but now I can’t cry even though I want to*)	-.17	.38	[-0.50, 0.21]
11. Feeling irritated (*I feel irritated all the time*)	.034	.86	[-0.34, 0.40]
12. Interest for others (*I have lost all of my interest in other people*)	-.19	.34	[-0.52, 0.19]
13. Decision making (*I can’t make decisions at all anymore*)	.018	.93	[-0.35, 0.38]
14. Feeling ugly (*I believe that I look ugly*)	.15	.43	[-0.23, 0.49]
15. Efforts (*I can’t do any work at all.I can’t do any work at all*)	*.32*	.09	[-0.053, 0.61]
16. Insomnia (*I wake up several hours earlier than I used to and cannot get back to sleep*)	-.11	.57	[-0.46, 0.27]
17. Fatigue (*I am too tired to do anything*)	**.50**	.0055	[0.17, 0.73]
18. Appetite (*I have no appetite at all anymore*)	.10	.61	[-0.28, 0.45]
19. Weight loss (*I have lost more than fifteen pounds*)	-.13	.49	[-0.48, 0.24]
20. Health concerns (*I am so worried about my physical problems that I cannot think of anything else*)	.28	.15	[-0.10, 0.58]
21. Libido (*I have lost interest in sex completely*)	-.18	.35	[-0.51, 0.20]

Bold values indicate significant correlations (p < .05).

### 3.3 Additional analyses

In additional analyses, we also investigated whether the group of patients exhibiting a self-focus attentional bias (i.e., focusing more on the self after vs. before recalling an episode of failure, n = 9) differed from those displaying self-focus avoidance (i.e., focusing less on the self after vs. before recalling an episode of failure, n = 20) in terms of demographic characteristics, psychiatric disorders, and pharmacological treatment (see **Table 2**). Overall, we found minimal differences between the two groups. It is noteworthy, however, that patients presenting a self-focus attentional bias after failure had significantly higher levels of depressive symptoms on the BDI scale (but not on the MADRS). Additionally, participants displaying the self-focus bias were marginally more likely to have a high suicide risk on the MINI (7/9 or 77%) compared to those presenting a self-focus avoidance bias (8/20 or 40%, p = .06).

**Table 2:** Comparisons of patients displaying self-focus attentional bias and avoidance attentional bias.

Characteristics	Self-focus attentional bias	Self-focus avoidance bias	Chi-Squared	t-test
Demographics				
Age	M = 39.5 (SD = 13.53)	M = 37.0 (SD = 13.51)	*N/A*	*t*(27) = – 0.46, *p* = .65
Gender	14 women, 6 men	9 women, 0 men	χ^2^ = 3.40, *p* = .065	*N/A*
Clinical Variables				
BDI	25.95 (SD = 11.45)	35.78 (SD = 12.38)	*N/A*	*t*(27) = 2.09, *p* = .046
MADRS	25.95 (SD = 6.57)	30.44 (SD = 6.84)	*N/A*	*t*(27) = 1.38*, p* = .18
Average number of attempted suicides	1.6 (SD = 1.63)	2.11 (SD = 1.83)	*N/A*	*t*(27) = 0.75, *p* = .46
Psychiatric Condition (MINI)				
Bipolar depression	2/20 = 10%	2/9 = 22.22%	*χ* ^2^ = 0.78, *p* = .38	*N/A*
Unipolar depression	14/20 = 70%	7/9 = 77.78%	*χ* ^2^ = 0.19, *p* = .67	*N/A*
Melancholic characteristics	11/20 = 55%	4/9 = 44.44%	*χ* ^2^ = 0.28, *p* = .60	*N/A*
Previous Suicide attempts	13/20 = 65%	7/9 = 77.78%	*χ* ^2^ = 0.47, *p* = .49	*N/A*
Previous depressive episodes	15/20 = 75%	7/9 = 77.78%	*χ* ^2^ = 0.026, *p* = .87	*N/A*
Suicide risk	18/20 = 90%	9/9 = 100%	*χ* ^2^ = 0.97, *p* = .33	*N/A*
Low suicide risk	8/20 = 40%	2/9 = 22.22%	*χ* ^2^ = 87, *p* = .35	*N/A*
Medium suicide risk	2/20 = 10%	0/9 = 0%	*χ* ^2^ = 97, *p* = .33	*N/A*
High suicide risk	8/20 = 40%	7/9 = 77.78%	*χ* ^2^ = 3.55, *p* = .06	*N/A*
Previous maniac episode	5/20 = 25%	1/9 = 11.11%	*χ* ^2^ = 0.73, *p* = .39	*N/A*
Panic attacks	3/20 = 15%	4/9 = 44.44%	*χ* ^2^ = 2.94, *p* = .086	*N/A*
Agoraphobia	13/20 = 65%	4/9 = 44.44%	*χ* ^2^ = 1.081, *p* = .30	*N/A*
Social phobia	4/20 = 20%	4/9 = 44.44%	*χ* ^2^ = 1.86, *p* = .17	*N/A*
OCD	3/20 = 15%	2/9 = 22.22%	*χ* ^2^ = 0.23, *p* = .63	*N/A*
PTSD	4/20 = 20%	2/9 = 22.22%	*χ* ^2^ = 0.019, *p* = .89	*N/A*
Alcoholism	4/20 = 20%	1/9 = 11.11%	*χ* ^2^ = 0.34, *p* = .56	*N/A*
Alcohol abuse	1/20 = 5%	1/9 = 11.11%	*χ* ^2^ = 0.36, *p* = .55	*N/A*
Substance addiction	1/20 = 5%	1/9 = 11.11%	*χ* ^2^ = 0.36, *p* = .55	*N/A*
Current psychotic syndrome	1/20 = 5%	1/9 = 11.11%	*χ* ^2^ = 0.36, *p* = .55	*N/A*
Mood disorder with psychotic characteristics	1/20 = 5%	2/9 = 22.22%	*χ* ^2^ = 1.99, *p* = .16	*N/A*
Bulimia	2/20 = 10%	2/9 = 22.22%	*χ* ^2^ = 0.78, *p* = .38	*N/A*
Generalized Anxiety	10/20 = 50%	5/9 = 55.56%	*χ* ^2^ = 0.077, *p* = .78	*N/A*
Pharmacological Treatment				
Antipsychotics	8/20 = 40%	3/9 = 33.33%	*χ* ^2^ = 0.12, *p* = .73	*N/A*
Levothyrox	3/20 = 15%	1/9 = 11.11%	*χ* ^2^ = 0.079, *p* = .78	*N/A*
Zopiclone	5/20 = 25%	1/9 = 11.11%	*χ* ^2^ = 0.73, *p* = .39	*N/A*
Benzodiazepine	16/20 = 80%	8/9 = 88.89%	*χ* ^2^ = 0.34, *p* = .56	*N/A*

## 4 Discussion

Depressed individuals typically demonstrate biased attention allocation toward negative stimuli, biased memory recall of negative stimuli, and biased negative interpretations of ambiguous situations (13, 34, 49–52). Depressive symptoms have been shown to correlate with higher self-focus (32, 53; see (54) for a meta-analysis). It has been postulated that depression might be characterized by a maladaptive self-focusing bias in failure situations (i.e., a tendency to direct one’s attention inward after experiencing failure; 12). In this study, we investigated whether clinical depression is linked to a self-focus pattern indicative of a maladaptive (as indicated by more self-focus following failure) self-focusing style and assessed how specific depressive symptoms might be associated to the bias.

Generally, our findings revealed fewer gazes directed towards the self after autobiographical recall of a failure experience, compared to before. Although this pattern contradicts the depressive self-focusing style hypothesis, it is largely consistent with self-focus avoidance observed in non-clinical samples (40, 55–57). This could suggest that the self-protective tendency to avoid self-awareness after failure is not entirely disrupted in depression, or that antidepressant medication effectively suppresses the debilitating self-focus attentional bias. Alternatively, this might provide evidence for a lack of attentional bias in depression, as emphasized in a study by Krings et al. (14).

However, approximately 30% of the patient group exhibited a self-focused attentional bias. Further analyses identified this maladaptive bias to be associated with specific depressive symptoms associated to failure sensitivity and self-blame: guilt, feelings of deserving punishment, self-hatred and self-blame. These findings suggest that a maladaptive self-focusing style might be confined to depressed patients presenting a certain profile regarding their relation to failure and the self. Moreover, patients who displayed a self-focused attentional bias post-failure, as compared to the remaining patients, also reported higher levels of depressive symptoms and a higher suicide risk, hence suggesting that the self-focus bias might be confined to severe forms of depression. These findings indicate that a self-focused attentional bias is linked to a more self-destructive pattern, which might have substantial clinical implications, as interventions targeting this cognitive bias could be particularly beneficial for this subgroup of patients.

The finding that only BDI scores, but not MADRS scores, predicted a higher self-focused attentional bias can be explained by the fundamental differences between these two scales. The BDI is a self-report inventory designed to capture a broad spectrum of depressive symptoms, including cognitive and affective components like guilt, self-dislike, and pessimism. These symptoms are intimately tied to the cognitive distortions and biases that characterize depression, making the BDI particularly sensitive to maladaptive thinking patterns such as a self-focused attentional bias. On the other hand, the MADRS is a clinician-administered scale that, while also assessing depression severity, places a greater emphasis on observable symptoms, including mood-related aspects like sadness and physical symptoms such as reduced appetite and lassitude. Although MADRS does include items related to pessimism and suicidal thoughts, it is less focused on the introspective, cognitive symptoms that are central to the BDI.

The current eye-tracking paradigm has limitations. First, gazing at the screen center may represent a strategy to minimize distance to the next target rather than self-directed attention, which could explain why dwell time is not a strong indicator of self-focus in this context (see 40). A pre- vs. post-test design, as used in this study, helps reduce such biases. However, including a control condition without a mirror surface could better differentiate between strategic gazing and self-directed attention. Using a within-participant design might increase participants’ awareness of the study’s true purpose, compromising its implicit nature. A between-participant design would preserve this implicit measure but reduce statistical power. For instance, while our paired t-test can detect an effect size of dz = .53, a between-participant design would require an effect size of d = 1.06 to achieve 80% power, making smaller effects harder to detect.

The relatively low sample size of this study already constitutes a limitation, which could have constrained our ability to detect significant effects. This could account for some of the marginally significant effects observed in this study. Unfortunately, small sample sizes are a common issue in clinical settings, limiting the generalizability of findings. Moreover, the sample was predominantly female. Although it was shown that depression was more prevalent among women, future studies might need to conduct replication in more balanced samples to assess the importance of gender on the observed findings. Regarding the task, although there were no feedbacks indicating “success” or “failure” for each trial, the nature of the lexical decision task might influence general feelings of failure. Future studies might integrate different task that would be less evaluative in order to avoid possible confounding effects from this aspect of the task. In addition, because we relied on a symptom-focused approach (i.e., computing correlations for each item of the BDI), false positive rates might have been inflated. Future replications are necessary to confirm the robustness of the present effects. Moreover, our sample was characterized by several comorbidities, such as anxiety, which could be confounding variables. It would be desirable to isolate the effect of each pathology, though this might pose a challenge, given the substantial comorbidity in depression. Lastly, it is possible that some effects were influenced by the pharmacological treatment patients were undergoing at the time of the study. Although it would be ideal, it is ethically unfeasible to replicate the present study in a sample of currently depressed patients not under treatment.

In conclusion, the present results suggest that, ceteris paribus, the self-focus attentional bias in depression might be weaker than expected based on existing literature. However, this particular bias might be indicative of the most severe forms of depression as indicated by a greater self-focus bias among patients characterized by a high suicide risk. In particular, high levels of symptoms reflecting self-blame and sensitivity to failure (guilt, punishment, self-hatred and self-blame symptoms) positively correlated with greater self-focus after vs. before recalling failure. Given the importance of aversive self-awareness, guilt and self-blame in suicide risk (55, 58), further investigations are warranted to understand the development of a depressive self-focusing style and strategies to mitigate it in clinical settings. Future research is needed to evaluate if targeting this bias could serve as an effective clinical intervention strategy.

## 5 Footnotes

(1) Inter-trial intervals used for the training block were: 490 ms, 566 ms, 677 ms, 754 ms, 1194 ms, 1480 ms, 3310 ms, 4237 ms, 4435 ms, 6531 ms, 7178 ms, 7281 ms. Inter-trial intervals used for each experimental block were: 325ms, 236 ms, 378 ms, 432 ms, 454 ms, 558 ms, 678 ms, 745 ms, 862 ms, 917 ms, 936 ms, 959 ms, 1040 ms, 1073 ms, 1117 ms, 1131 ms, 1235 ms, 1256 ms, 1310 ms, 1399 ms, 3197 ms, 3272 ms, 3277 ms, 3404 ms, 4079 ms, 4639 ms, 5527 ms, 5756 ms, 6195 ms, 6245 ms, 6352 ms, 6452 ms, 7204 ms, 7934 ms, 8480 ms, 8485 ms.

(2) As requested by an anonymous reviewer, a median split was performed to assess self-focus avoidance when comparing individuals with high BDI scores (i.e., BDI scores greater than the median score of the sample) and low BDI scores (i.e., BDI scores smaller than the median score of the sample). A student t-test revealed that patients with high BDI scores displayed higher self-focus after the manipulation of failure (M = -0.53, SD = 8.04) than participants with low BDI scores (M = -7.43, SD = 7.98), t(27) = -2.32, p = .028, d = -0.86, 95%CI[-1.62, -0.09]. We warmly thank the anonymous reviewer for their suggestion.

## Data Availability

The datasets presented in this study can be found in online repositories. The names of the repository/repositories and accession number(s) can be found below: https://osf.io/94y7v/.
