# Supplementary material for: Depressive self-focus bias following failure: an eye-tracking study among individuals with clinical depression
Source: Front Psychiatry. 2024 Oct 1;15:1459831. doi: 10.3389/fpsyt.2024.1459831 (PMC11473297; doi:10.3389/fpsyt.2024.1459831)

Supplementary Material

# Additional analyses on guilt and shame

We used a modified version of the State Shame and Guilt Scale (SSGS, Marschall et al., 1994) to assess shame- and guilt-proneness. The scale was modified to evaluate a stable disposition. In order to assess how guilt-proneness scores qualified this effect of failure manipulation, we computed the registered linear regression on the DV using guilt-proneness scores as a moderator.

We failed to detect a significant effect of guilt-proneness scores at the alpha threshold of 5%, t(28) = 2.00, p = .055, eta2 = .13. In an exploratory approach, we assessed how shame-free guilt (i.e., the part of guilt that is not explained by shame, computed using residual guilt resulting from the regression predicting guilt by shame scores, e.g., Tangney et al., 1992, see Tangney & Dearing, 2002 for a short discussion) might influence self-focus avoidance following failure. Shame-free guilt significantly qualified self-focus following failure, t(27) = 2.23 , p = .034, eta2 = .16. (see figure 2). The greater the shame-free guilt score, the less self-focus avoidance was displayed.

**Figure 2.** *Effect of guilt scores on self-focus avoidance indicated through* ***number of entries in the AOI*** *(the smaller the score, the greater self-focus avoidance; a score of zero indicates an equal amount of entries after vs before the failure manipulation).*


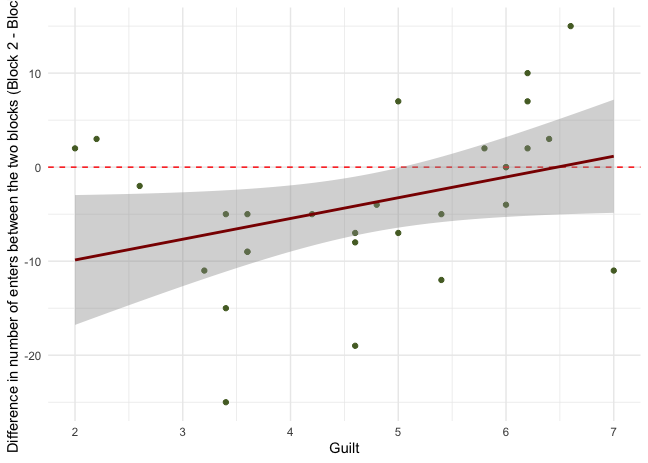


# Additional Analyses on Dwell Time

*Notice of caution*

Dwell time and number of saccades are negatively correlated in our sample, *Spearman’s rho* = – .33, *p* = .006 (on 58 pairs of observations). In fact, these criteria share a peculiar non-linear relationship as described in figure 1, 2, and 3 illustrating their relation in previous and current data (Monéger et al., 2022; Monéger et al., in preparation). Studies using a similar protocol show that dwell time is insensitive to failure manipulation. In contrast, number of saccades in the AOI was consistently associated to variations associated to failure manipulations in the registered directions. Therefore, interpretation of the dwell time results should be cautiously done.

**Figure 1**. Relation between dwell time and number of saccades in the AOI in Monéger et al. (2022)


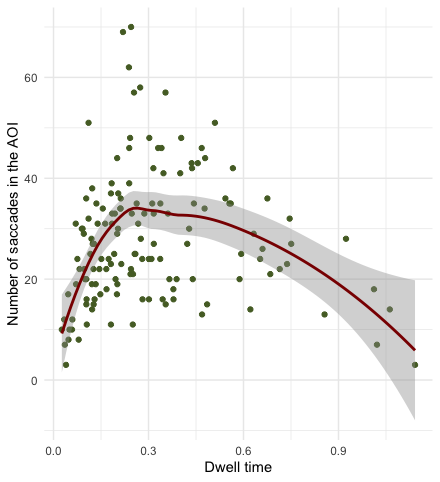


**Figure 2**. Relation between dwell time and number of saccades in the AOI in Monéger et al. (in preparation)


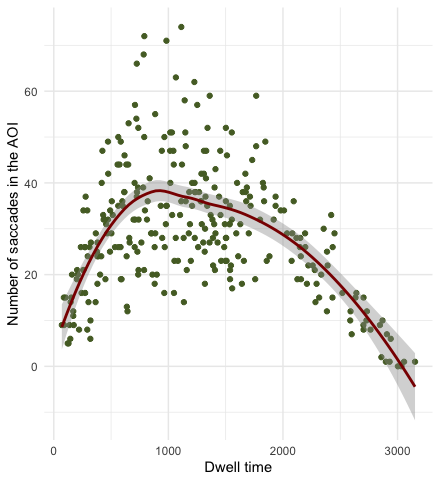


**Figure 3**. Relation between dwell time and number of saccades in the AOI in the current study


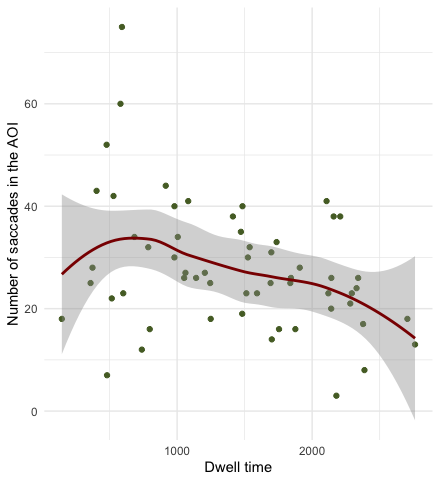


We performed similar analyses on dwell time and failed to find evidence for self-focus nor self-focus avoidance among depressed, *t*(28) = 1.07, *p* = .29, *Cohen’s d_z_* = – 0.20, *95%CI* [– 0.58, 0.17]. However, inconsistent with the maladaptive self-focus account of depression, BDI scores did predict greater self-focus *avoidance* after (vs before) the autobiographical recall, *t*(27) = – 2.22, *p* = .035, $\eta_{p}^{2}$ = .15, *95%CI* [.00,.40]. The more individual scored high on depression severity, the more self-focus avoidance was displayed after the autobiographical recall.

In order to better understand what specific aspect of depression severity interacted with the autobiographical recall to predict biased self-focus, we once again used relevant items of the BDI scale as predictors in independent regression models using difference in time spent in the AOI after vs. before the autobiographical recall. The effect was for a large part driven by scores to the item assessing sense of failure, *t*(27) = – 3.96, *p* < .001 $\eta_{p}^{2}$ = .37, 95%CI [.10, .58]. The guilt symptom of depression failed to significantly predict self-focus, *t*(27) = – 1.94, *p* = .063, $\eta_{p}^{2}$ = .12, *95%CI* [.00,.37]. However, punishment significantly correlated with self-focus, *t*(27) = – 2.70, *p* = .012, $\eta_{p}^{2}$ = .21, *95%CI* [.01, .46] such that the higher the scores on the punishment symptom, the more self-focus avoidance was displayed among depressed patients. Finally, self-accusation also failed to reach significance although showing a similar trend than the aforementioned symptoms, *t*(27) = – 1.97, *p* = 0.059, $\eta_{p}^{2}$ = .13, *95%CI* [.00, .37].

**Figure 4**. Correlation between the item assessing sense of failure and the difference between dwell time spent on the AOI in the two blocks (Block 2 – block 1)


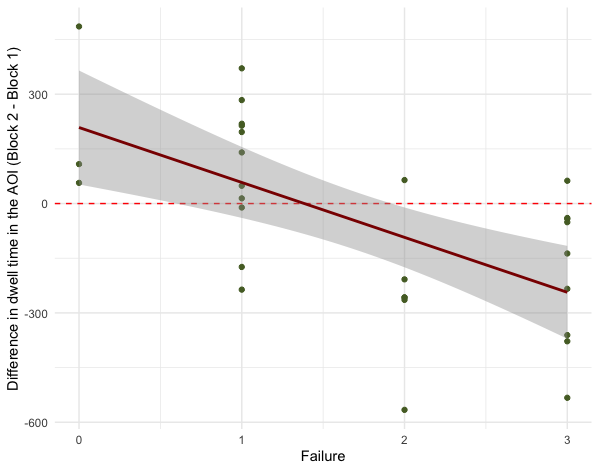

Supplement: Supplementary file 1 [file DataSheet1.docx]
